# Mycobacterial Spindle Cell Pseudotumor in an 18‐Year‐Old Female With Congenital HIV: A Case Report and Literature Review

**DOI:** 10.1155/carm/1612986

**Published:** 2025-12-17

**Authors:** Zahra Aminparast, Zeinab Shakibaee Fard, Amirmohammad Khodaei

**Affiliations:** ^1^ Department of Pathology, Clinical Research Development Center, Imam Reza Hospital, Kermanshah University of Medical Sciences, Kermanshah, Iran, kums.ac.ir; ^2^ Student Research Committee, Medical School, Kermanshah University of Medical Sciences, Kermanshah, Iran, kums.ac.ir

**Keywords:** acid-fast bacilli, CD68, HIV, immunocompromised, inflammatory pseudotumor, mycobacterial spindle cell pseudotumor, *Mycobacterium tuberculosis*

## Abstract

Mycobacterial spindle cell pseudotumor (MSP) is an uncommon granulomatous inflammation often found in immunocompromised patients, especially those with HIV. This case report discusses an 18‐year‐old female with congenital HIV, who presented with severe epigastric pain, nausea, and noteworthy lymphadenopathy. Imaging studies identified lobulated masses, and a core needle biopsy confirmed the diagnosis of MSP, revealing spindle‐shaped histiocytes and acid‐fast bacilli. Additionally, we reviewed nine MSP cases documented since 2017.

## 1. Introduction

Mycobacterial spindle cell pseudotumor (MSP) is a form of granulomatous inflammation that was initially described by Wood et al. in 1985 in the skin of a patient with cardiac transplantation [1]. MSP is frequently observed in individuals who are immunocompromised, including those with human immunodeficiency virus (HIV) infection [2].

MSP can result from both tuberculous and nontuberculous mycobacteria (NTM). The majority of documented cases of MSP are linked to the *mycobacterium avium complex* (MAC) [3].

It is an uncommon condition that is diagnosed by the growth of spindle‐shaped histiocytes that contain intracytoplasmic acid‐fast bacilli or as an enhancing lesion on computed tomography (CT) scan. Recognizing this benign condition is crucial because the differential diagnosis is broad Kaposi’s sarcoma (KS) and other mesenchymal tumors [4–6].

## 2. Case Presentation

An 18‐year‐old female patient with congenital HIV presented with severe epigastric pain radiating to her back, along with nausea and five episodes of bilious vomiting over the past two days. She reported a weight loss of 1‐2 kg in the last month and had a family history of HIV in both parents.

Physical examination revealed generalized lymphadenopathy. Her vital signs were stable, and she was on tenofovir disoproxil fumarate (TDF), emtricitabine, and dolutegravir.

Laboratory results showed hemoglobin at 10.2 g/dL, hematocrit at 30.5%, and neutrophilia (88%), with a WBC count of 19.6 × 10^3^/μL. Serum albumin was low at 2.8 g/dL, and sodium levels were also low at 134 mmol/L. Tests for HBsAg, HCV, and IgG toxoplasma antibodies were nonreactive.

Subsequent abdominal ultrasound revealed a lobulated mass‐like area measuring 74 × 104 mm, which was noted in the epigastric region. An ultrasound of the neck and axillary regions showed multiple reactive lymph nodes.

**Figure 1:**
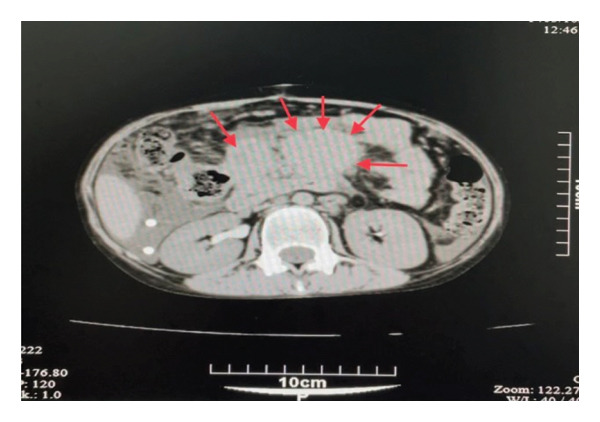
Axial contrast‐enhanced abdominal CT showing a conglomerate cluster of enlarged mesenteric lymph nodes (red arrows). The nodes appear homogenous and form a bulky mesenteric mass consistent with mycobacterial spindle cell pseudotumor*.*

A CT scan of the abdomen and pelvis showed splenomegaly (146 mm) with normal parenchymal density, along with significant mesenteric lymphadenopathy, having a maximum short‐axis diameter (SAD) of 14 mm. Two large masses in the epigastric region measured 57 × 46 mm and 54 × 40 mm, raising suspicion for conglomerate lymph nodes (Figure 1), and there was concern for lymphoma or opportunistic infections secondary to HIV. A noncontrast spiral CT of the neck and chest was unremarkable. Subsequently, a core needle biopsy of the abdominal lymph nodes was performed, obtaining a fixed specimen in formalin comprising several pieces of creamy‐colored, needle‐shaped tissue measuring 0.3 × 0.3 × 0.2 cm.

The hematoxylin and eosin (H&E)‐stained sections demonstrated abundant spindle cells as the predominant cell population, arranged in characteristic storiform or fascicular architectural patterns (Figures 2(a) and 2(b)). The lesion contained scattered foamy and epithelioid histiocytes, with occasional granulomas and foci of necrosis, resulting in significant effacement of the background tissue architecture. Granulomas and multinucleated giant cells were observed but represented infrequent findings.

Figure 2(a) Hematoxylin and eosin‐stained sections showing predominance of spindle cells in storiform and fascicular patterns with scattered foamy and epithelioid histiocytes. Minimal atypia, low mitotic activity, and mixed inflammatory infiltrate are observed (× 10) (photo by Dr. Aminparast, pathologist). (b) Hematoxylin and eosin‐stained sections showing predominance of spindle cells in storiform and fascicular patterns with scattered foamy and epithelioid histiocytes. Minimal atypia, low mitotic activity, and mixed inflammatory infiltrate are observed (× 40) (photo by Dr. Aminparast, pathologist).

**Figure:**
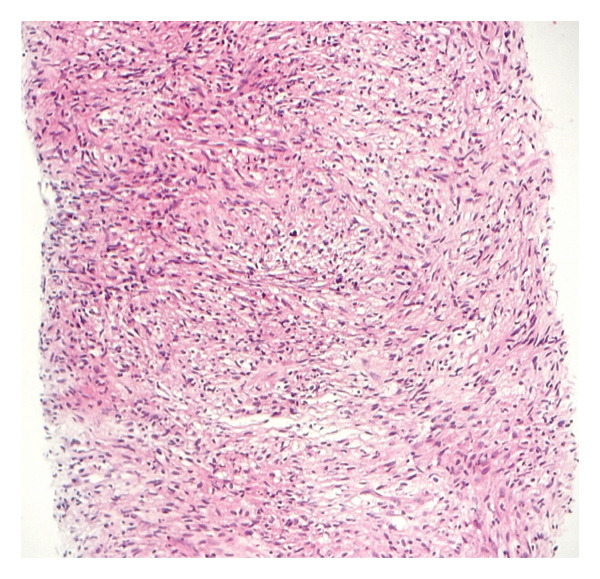
(a)

**Figure:**
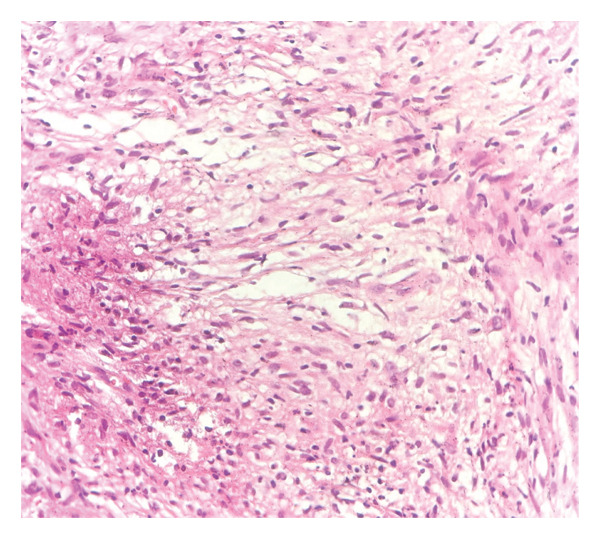
(b)

The spindle cells exhibited an expanded tumor‐like growth pattern with minimal cellular atypia and low mitotic activity, maintaining well‐defined boundaries. Notably, a proportion of epithelioid histiocytes displayed foamy cytoplasmic changes, morphologically resembling the adjacent foamy histiocytic infiltrate. These foamy histiocytes were characteristically distributed along the periphery of the predominantly spindled lesion.

Microscopic examination revealed a mixed inflammatory infiltrate composed of lymphocytes admixed with plasma cells, neutrophils, and eosinophils. Importantly, no atypical lymphoid cells or evidence of carcinoma were identified.

**Figure 3:**
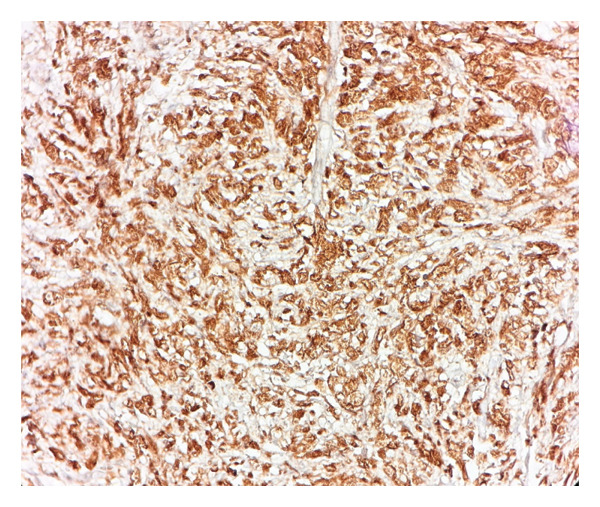
Immunohistochemical staining showing diffuse CD68 positivity, confirming the histiocytic lineage of the proliferating cells (× 40) (photo by Dr. Aminparast, pathologist).

**Figure 4:**
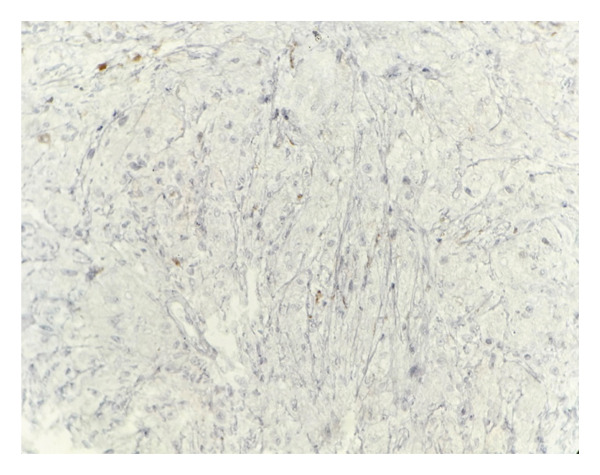
Immunohistochemical staining for S‐100 highlighting scattered spindle cells and intracytoplasmic bacilli (× 40) (photo by Dr. Aminparast, pathologist).

Immunohistochemical studies demonstrated diffuse CD68 positivity, confirming the histiocytic lineage of the proliferating cells (Figure 3), while S‐100 protein highlighted scattered spindle cells and intracytoplasmic bacilli (Figure 4).

**Figure 5:**
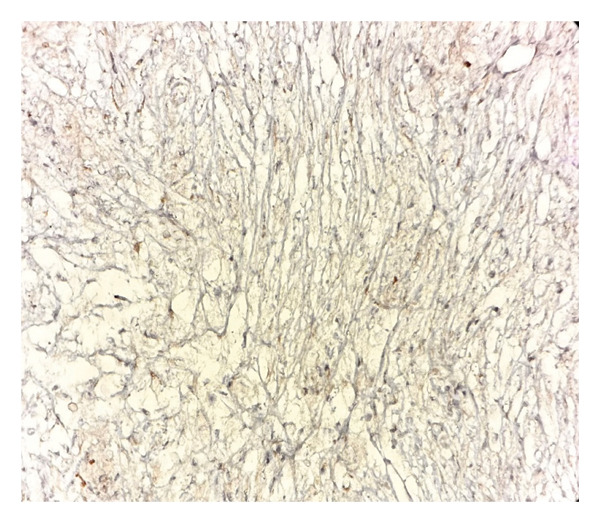
Immunohistochemical staining showing negativity for desmin in the proliferating cells (× 40), supporting the nonmuscle lineage of the lesion (photo by Dr. Aminparast, pathologist).

**Figure 6:**
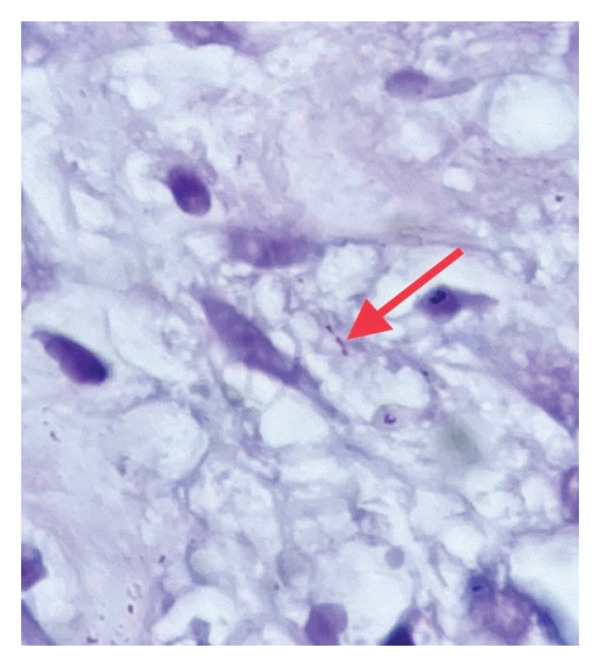
Acid‐fast bacilli (AFB) staining (× 100) demonstrating *Mycobacterium* within the lesional tissue (red arrow), confirming the presence of mycobacterial infection (photo by Dr. Aminparast).

The lesion was negative for cytokeratin (CK), desmin (Figure 5), and human herpesvirus 8 (HHV8). Molecular studies showed negative PCR results for *Mycobacterium tuberculosis*, though acid‐fast staining revealed rare beaded bacilli (Figure 6). These histopathological features—particularly the spindle‐shaped histiocytes containing acid‐fast bacilli—are diagnostic of MSP.

The patient later developed significant abdominal ascites, requiring the placement of an intervention drain for fluid removal, as well as an excisional lymph node biopsy. Approximately 4 L of tense ascitic fluid were drained under ultrasound guidance, with samples submitted for testing.

A hematology consultation was requested due to the lymph node pathology findings, which revealed no evidence of malignancy. It was recommended that antituberculosis treatment continue.

The patient’s condition subsequently improved, with the ascitic fluid transitioning from chylous to clear, and her overall status stabilized, although continued monitoring for ongoing ascites was necessary.

## 3. Discussion

MSP, a type of granulomatous inflammation, represents a complex interaction between mycobacterial infection and host immune responses, particularly in individuals with immunocompromising conditions like HIV infection and patients undergoing immunosuppressive therapy [7]. In our case, the patient was born with congenital HIV.

We conducted a review study of MSP case reports since 2017 that provided data almost similar to a study conducted between 1950 and 2017 by Sfeir et al. [8]. In our review (Table 1), we identified nine cases. The median age of patients was 49.5 years (with an age range of 26–76 years), and all patients had underlying disease. Similar to the findings of Sfeir et al. [8], the majority of cases were male and had an underlying HIV condition. Although several anatomical locations have been reported, the lymph nodes were the most commonly affected site. *Mycobacterium avium* was the most frequently isolated organism with secondary connections to *Mycobacterium tuberculosis*. Most patients received antibiotic treatment and exhibited a favorable prognosis.

**Table 1:** Previous cases regarding mycobacterial spindle cell pseudotumor.

	Year	Age/gender	Medical history	Initial presentation	Physical examination	Anatomic site and size	IHC and histochemical findings	CD4 counts (cells/μL)	Microbiological findings criteria	Imaging findings	Treatment	Follow‐up
Pascual et al. [9]	2018	50M	Immunodeficiency conditions, diabetes	Genital lesions, slight pruritus	Yellowish papules on shaft and foreskin	Penis and foreskin (1 × 1 cm ulcer)	CD31+, lysozyme+, CD68+, factor XIIIa+, D2‐40+ acid‐fast bacteria on Ziehl‐Neelsen	Not specified	*Mycobacterium avium complex*	Not specified	Azithromycin, ethambutol	Aggressive nodal anaplastic large T‐cell lymphoma diagnosed after 2 months; patient died shortly thereafter, no autopsy performed
Krause and Findeis [10]	2020	42 F	HIV	Fever, chills, night sweats, weight loss, abdominal fullness	Unremarkable	Large abdominal mass	AFB+	56	*M. avium complex*	Imaging studies revealed a large abdominal mass and splenomegaly	Not specified	Not specified
Lim et al. [5]	2021	48 M	T‐cell leukemia, immunocompromised status	Fever, lethargy	Unremarkable	Abdominal mass (5.3 cm)	CD163+, AFB+, S‐100 immunostain focally highlighted spindle cells and intracytoplasmic bacilli	Not specified	Positive for numerous acid‐fast bacilli	Computed tomography scan revealed a 5.3‐cm upper abdominal wall soft tissue mass with xiphoid process erosion	Not specified	Not specified
Singh et al. [11]	2021	36 M	HIV	Generalized weakness, fever, dizziness on ambulation	Palpable bilateral axillary lymphadenopathy	Bilateral axillary and left supraclavicular lymphadenopathy, enlarged mesenteric nodes	CD68+	5	*M. avium*	Noncontrast CT of the chest, abdomen, and pelvis revealed, left supraclavicular adenopathy, multiple enlarged mesenteric lymph nodes, mildly enlarged retroperitoneal lymph node, hepatosplenomegaly	Ethambutol, azithromycin, rifabutin	Improved
Mann et al. [12]	2023	4M	HIV, DLBCL	Worsening hypermetabolic lesions. Worsening hypermetabolic lesions on PEn PET/CT	Small supraclavicular lymphadenopathy	Supraclavicular (small)	Abundant mycobacterial organisms	< 35	*M. avium complex*	Not specified	Rifampin, azithromycin, ethambutol	Improved
Argyris and Wakely [13]	2023	74M	Oncology	Nasal polyp, chronic sinusitis	Unremarkable	Left nasal cavity (0/5 cm)	CD68+, AFB+, CD3 highlighted scattered lymphocytes	Not specified	Not specified	Not specified	Excision	No recurrences during 24‐month follow‐up
Kurosawa et al. [14]	2023	60 F	SLE	Tumor on lower right leg	Nodule smooth‐surfaced	Lower right leg, (20 × 18 mm)	AFB+	Not specified	Positive PCR for *M. avium complex, M. genavense* identified by genetic analysis	Whole‐body CT scan showed no lesions suspected of mycobacterial infection in lungs or liver	Combination therapy of clarithromycin, ethambutol, and rifampicin	Lost to follow‐up after the initial treatment regimen was recommended
Arthur et al. [4]	2023	76M	HIV	New rash over legs and arms	Erythematous tender nodule on the leg	Right distal pretibial (2 cm)	CD68+, AFB+, CD68, CD3, and CD20 stains confirmed the lymphohistiocytic nature of the lesion	80	*M. tuberculosis*	Not specified	Not initiated	Lost to follow‐up before treatment
Li et al. [7]	2023	26 M	HIV	Fever, vomiting, diarrhea, progressive abdominal pain	Unremarkable	Terminal ileum, ileocecum, ascending colon, transverse colon, and descending colon	CD68+, AFB+	47	*M. tuberculosis complex*	Abdominal CT showed thickened intestinal wall, local effusion, and multiple cystic nodules. Colonoscopy revealed multiple nodular lesions in various segments of the colon	Isoniazid, rifampicin, pyrazinamide, and ethambutol (due to a rash, pyrazinamide and ethambutol were replaced by levofloxacin)	Improved

The pathophysiology is not fully understood but various hypotheses suggest it may involve host immune responses or part of the inflammatory cytokine response to the pathogen, leading to local proliferation of spindle cells and mycobacterium colonization [2]. MAC can evade macrophage defense mechanisms, allowing it to replicate intracellularly, yet it is sensitive to CD4 and natural killer cells. Therefore, it is more common in individuals with immune deficiencies, much like our patient, who presented with a low CD4 count [12].

The histologic differential diagnosis for MSP is broad and includes both neoplastic and reactive conditions [10]. Key considerations include KS, inflammatory myofibroblastic tumor, nodular fasciitis, benign fibrous histiocytoma, follicular dendritic cell sarcoma, spindle cell melanoma, and histiocytic/dendritic cell neoplasms. Other potential mimics encompass myogenic and neural tumors (e.g., leiomyosarcoma and neurofibroma), vascular neoplasms (angiosarcoma), mesenchymal tumors (solitary fibrous tumor and low‐grade fibromyxoid sarcoma), and infectious or reactive lesions (inflammatory fibroblastic proliferations and other infectious pseudotumors). Distinguishing MSP relies on histomorphology, immunohistochemistry, and microbiological studies to rule out these entities [2, 4].

Although MSP is benign, it poses significant diagnostic challenges due to histological similarities with malignant tumors, particularly KS. KS, similar to MSP, is associated with HIV and often affects similar sites, usually lymph nodes [2].

The diagnosis of MSP relies on histomorphology, immunohistochemistry, and microbiological studies to rule out other entities. Histopathologic examination of tissue samples with acid‐fast staining can confirm the presence of mycobacteria. Close morphologic evaluation often reveals subtle signs of an infectious etiology, including admixed neutrophilic inflammation and ill‐defined granulomas. Another helpful feature is the presence of foamy histiocytes, which resemble those seen in other nontuberculous mycobacterial infections. These foamy histiocytes are typically located at the periphery of the spindle cell lesions [15].

The characteristic features of MSP include a lack of encapsulation and a nodular cellular proliferation of spindle‐shaped cells, which display an appearance that is bland and arranges in a haphazard, storiform, or vaguely fascicular pattern [13]. The spindled histiocytes are notable for having finely granular eosinophilic cytoplasm and nuclei that are either rounded or elongated, with vesicular chromatin. These spindle cells are typically observed surrounding small blood vessels and are associated with varying degrees of inflammatory infiltrate [5].

Immunohistochemically, MSP cells commonly express histiocytic and macrophagic markers, including CD68, CD11c, CD163, and CD204, as well as S‐100. Our sample staining was also positive for CD68 and S‐100. Additionally, they exhibit a low Ki67 proliferation index, which aids in differentiating MSP from malignant processes. In contrast, KS cells are positive for endothelial markers such as CD31 and CD34 [6, 13].

Reported treatments include antimycobacterial therapy, surgical removal, and a combination of antimycobacterial agents plus surgery, or no treatment at all. Surgery may be necessary for pressure symptoms or diagnostic clarification; however, the majority of cases typically respond well to medication alone [11].

The infrequent occurrence of MSP, combined with its unusual clinical presentations, frequently results in diagnostic delays. As a result, it is important to include MSP in the differential diagnosis when evaluating spindle cell lesions in patients with compromised immune systems.

## 4. Patient Perspective and Recommendation

Formal ascertainment of the patient’s personal perspective was precluded by the exigent need for diagnostic and therapeutic interventions during the acute phase of her illness. However, the clinical course highlights the considerable psychological burden that a provisional diagnosis of malignancy imposes on a young patient. The eventual confirmation of a treatable infectious pseudotumor, following a comprehensive histopathological workup, provided considerable relief and underscores the critical importance of a meticulous diagnostic evaluation in mitigating patient anxiety.

This case highlights the critical importance of considering infectious pseudotumors like MSP in the differential diagnosis of spindle cell lesions in immunocompromised patients, particularly those with HIV. The key lesson is the necessity of distinguishing this benign entity from malignancies like KS, which is achieved through an appropriate immunohistochemical panel (e.g., CD68 positivity and HHV‐8 negativity) and acid‐fast staining to identify the causative bacilli. Fortunately, the condition typically responds well to targeted antimycobacterial therapy once correctly diagnosed.

The main recommendation is to enhance awareness of MSP among both clinicians and pathologists. Close collaboration between these specialties is essential to avoid misdiagnosis as cancer, prevent unnecessary patient anxiety, and ensure the prompt initiation of appropriate antimicrobial treatment.

## Ethics Statement

This manuscript does not include personal or medical details about any identifiable individual. The patient’s adoptive mother provided consent for the writing and publication of this article.

## Consent

Apart from age and gender, no identifying details were included in the manuscript. The patient’s adoptive mother provided written consent for the utilization of their hospital records and imaging as per the journal’s patient consent guidelines.

## Conflicts of Interest

The authors declare no conflicts of interest.

## Author Contributions

Zahra Aminparast: conception, investigation, resources, supervision, and writing–original draft. Zeinab Shakibaee Fard: acquisition and analysis of data, investigation, software, supervision, drafting manuscript, and writing–review and editing. Amirmohammad Khodaei: investigation, project administration, software, drafting manuscript, and writing–review and editing.

## Funding

This research was not funded by any particular grants from public, commercial, or not‐for‐profit organizations.

## Data Availability

The data that support the findings of this study are available on request from the corresponding author. The data are not publicly available due to privacy or ethical restrictions.
